# Unicompartmental knee arthroplasty vs. high tibial osteotomy for medial knee osteoarthritis (UNIKORN): a study protocol of a randomized controlled trial

**DOI:** 10.1186/s13063-023-07263-7

**Published:** 2023-04-05

**Authors:** Juuso Siren, Lasse Rämö, Mikko Rantasalo, Olli Komulainen, Noora Skants, Aleksi Reito, Jussi Kosola, Jan Lindahl

**Affiliations:** 1 Department of Orthopaedics and Traumatology, University of Helsinki and Helsinki University Hospital, Topeliuksenkatu 5, 00260 Helsinki, Finland; 2 Department of Anaesthesiology, Intensive Care and Pain Medicine, Peijas Hospital, HUS Helsinki University Hospital, Sairaalakatu 1, 01400 Vantaa, Finland; 3 Department of Orthopaedics and Traumatology, Tampere University Hospital, Elämänaukio 2, 33520 Tampere, Finland; 4 Department of Orthopaedics and Traumatology, Kanta-Häme Central Hospital, Ahvenistontie 20, 13530 Hämeenlinna, Finland

**Keywords:** High tibial osteotomy, Unicompartmental knee arthroplasty, Intention to treat, Superiority trial, Knee osteoarthritis, Activity monitoring, Randomized controlled trial

## Abstract

**Background:**

Medial knee osteoarthritis (OA) is a common health problem resulting in knee pain and limiting patients’ physical activity. After failed conservative treatment, unicompartmental knee arthroplasty (UKA) and high tibial osteotomy (HTO) are possible surgical treatment options for this condition. There is a paucity of high-quality evidence in the literature comparing objective and subjective outcomes of these procedures. Also, there is no common agreement on whether these procedures provide comparable results in late-stage medial knee OA patients.

**Methods:**

We will perform a prospective randomized controlled trial comparing HTO and UKA in patients with late-stage medial knee OA. 100 patients with isolated medial knee OA (KL III–IV) are assigned to either UKA (*n* = 50) or HTO (*n* = 50) procedure in patients 45–65 years of age. Our primary outcome will be KOOS_5_ at one year postoperatively. Secondary outcomes include OARSI physical assessment, length of stay, wearable activity watch, radiographs (OA progression according to Kellgren-Lawrence classification), patient-reported outcomes (KOOS subscales, pain visual analog scale [VAS], Lysholm, and Oxford knee scores), and adverse events (conversion to total knee arthroplasty, surgery-related complications, need for revision surgery) outcomes. Our hypothesis is that neither of the interventions is superior as measured with KOOS_5_ at 12 months.

**Ethics and dissemination:**

The institutional review board of the Helsinki and Uusimaa Hospital District has approved the protocol. We will disseminate the findings through peer-reviewed publications.

**Trial registration:**

ClinicalTrials.gov/TooloH NCT05442242. Registered on 7/1/2022.

**Supplementary Information:**

The online version contains supplementary material available at 10.1186/s13063-023-07263-7.

## Introduction

Knee osteoarthritis (OA) is a common medical condition negatively affecting patients’ everyday lives and creating economic burden to societies [1–3]. Knee OA is often related to varus deformity and has various treatment options [4]. After failed nonsurgical care, total knee arthroplasty (TKA) has been the gold-standard treatment. Although TKA has proven superiority over nonsurgical care, [5] around 20% of patients are not satisfied with TKA [6–8]. In addition to TKA, unicompartmental knee arthroplasty (UKA) and high tibial osteotomy (HTO) are widely accepted treatment options in isolated unicompartmental OA [9]. Also, after failed UKA or HTO there is a possibility to convert to TKA.

In UKA, the damaged cartilage of the affected compartment is replaced by intra-articular implants. In a recent multicenter randomized controlled trial (RCT), UKA resulted in similar clinical outcomes and similar number of reoperations and complications compared with TKA. The cost-effectiveness of UKA was superior to TKA [10]. It has been estimated that 25–48% of knee OA patients would be suitable for UKA [4, 11]. However, the UKA rate has varied between 8 and 12.2% in different countries [12]. This suggests that UKA should be considered more often in patients with isolated medial compartment OA.

In HTO, the mechanical axis of the lower limb is corrected using an extra-articular osteotomy to shift weight towards the healthy lateral compartment in patients with medial knee OA and varus malalignment. The osteotomy can be performed with open or closed wedge technique [13]. While different complication profiles have been described, similar outcomes of these two methods have been reported [14]. Traditionally, HTO has been considered an option for younger active patients with a less severe OA. However, there is a paucity of high-quality evidence in the literature to support this assumption and in recent studies, HTO has been used in late-stage OA with good long-term results [15].

Recently, Jin et al. reported the first long-term follow-up results comparing HTO with UKA [16]. They found that WOMAC scores were superior in the UKA group but found no other significant differences between the groups in survival rates, complication rates, or OA progression. This study was performed using Propensity score matching (PSM) from a retrospective data.

To our knowledge, there are no previous RCTs comparing UKA with HTO in the treatment of late-stage medial knee OA. To fill this evidence gap, we designed this RCT to compare HTO and UKA in patients with medial late-stage OA of the knee with the following hypotheses.

1. Null hypothesis is that there is no clinically significant difference between HTO and UKA in the primary outcome measure (The Knee Injury and Osteoarthritis Outcome Score composite, KOOS_5_) at 12 months postoperatively.
2. There is no significant difference in rehabilitation time after surgery between HTO and UKA as measured with KOOS_5_ at follow-up timepoints.
3. We expect no progression in lateral compartment OA in imaging studies during follow-up.

## Methods and analyses

### Study setting

This study is a randomized controlled, pragmatic single-center, parallel-group, 1:1 superiority trial. Our goal is to compare the effect of HTO and UKA in patients with late-stage medial knee OA. The study will take place in Helsinki University Hospital. Approximately 50 UKAs and 50 HTOs are performed in our hospital every year.

### Eligibility criteria

Orthopedic surgeons identify potential study participants from the referrals to outpatient clinics. Patients are scheduled for an outpatient visit to a surgeon member of the study group. Patients aged between 45 and 65 years with primary medial unicompartmental knee OA (Kellgren-Lawrence [KL] III–IV) [17] with varus deformity of 4 degrees or more can participate in the study. The patients are diagnosed according to clinical and radiologic examinations including MRI, weight-bearing knee, and mechanical axis radiographs. The patients meeting the inclusion criteria are asked to participate in the study. Written information and a consent form are given to the patient at the outpatient clinic. If the patient is willing to participate in the trial, they are scheduled for an appointment to the outpatient clinic once more for randomization and baseline measures. The detailed inclusion and exclusion criteria are shown in Table 1.

**Table 1:** Inclusion and exclusion criteria used in the randomized controlled trial

**Inclusion criteria**
1. Symptomatic (more than 6 months) tibiofemoral medial joint arthrosis not responding to conservative treatment
2. Range of motion in clinical exam: extension 5° or less, flexion 120° or more
3. KL grade III–IV arthrosis in the medial compartment of the tibiofemoral joint
4. Modified Outerbridge grade III–IV arthrosis in MRI [18]
5. Varus alignment in mechanical axis ≥ 4°
6. Medial proximal tibial angle (MPTA) < 90°
7. Patient accepts the treatment options: HTO or UKA
8. Age 45–65 years
**Exclusion criteria**
1. Arthrosis in the lateral compartment of the tibiofemoral joint more than Modified Outerbridge classification grade I in MRI
2. Arthroscopic arthrosis more than grade I (Modified Outerbridge classification) in the lateral TF joint and/or meniscus tear or previous partial resection of meniscus
3. Instability due to ACL, PCL, MCL, or LCL insufficiency
4. Post-traumatic arthrosis
5. Inflammatory arthritis
6. Significantly impaired ability to co-operate for any reason (substance abuse, mental disorder, dementia)
7. Malignancy
8. Insulin-dependent diabetes
9. Previous surgery for instability of the knee joint (patellar instability, cruciate ligaments, MCL or LCL surgery)
10. Obesity (BMI > 35)
11. Unable to speak or read fluently either Finnish or Swedish (due to language used in data forms)
12. Contraindication for MRI

### Interventions

After recruitment, the patients are scheduled an appointment with a physical therapist and an individual training program is introduced. Patients are randomized to either HTO or UKA in 1:1 ratio. If the patients’ symptoms are reduced markedly before the operative treatment (1–4 months post randomization) a possibility for drop-out is offered. Both UKA and HTO operations are performed by experienced surgeons with at least 50 operations of UKA or HTO in total.

### Surgical technique

#### HTO — high tibial osteotomy (open wedge)

An open wedge HTO is a standard approach in our clinic and thus used as the surgical technique in this study. Weight-bearing mechanical axis radiograph is used to plan the desired correction. An arthroscopy of the knee is performed first to determine the degree of OA. Arthrosis of the lateral and medial TF joint is graded by the modified Outerbridge classification [18, 19]. Patients with lateral OA grade II or greater are excluded from the study at this point. Cruciate ligaments and menisci are also evaluated in the arthroscopy.

A medial high tibial open wedge osteotomy is performed using a diagonal incision just cranial to pes anserinus tendons or a medial longitudinal incision. Pes anserinus tendons are retracted distally and the superficial medial collateral ligament is exposed and incised at the level of the planned osteotomy. Two parallel K-wires are inserted under image intensifier to mark the osteotomy level. Patellar tendon is protected and a biplanar osteotomy line is created using an oscillating saw and osteotomes leaving the lateral cortex intact and the tibial tubercle to the distal side of the osteotomy. Thin osteotomes are used to open the osteotomy after sawing to achieve the desired correction (Fujisawa point) [20]. The osteotomy site is secured using a medial locking plate (TomoFix®, DePuy Synthes, Raynham, MA, USA). The wound is closed in a standard manner. Local infiltration analgesia is used for all patients. An elastic bandage is applied. Weight-bearing as tolerated is allowed immediately after the surgery.

#### UKA — unicompartmental knee arthroplasty

Oxford® Partial Knee (Zimmer Biomet, Warsaw, IN, USA) arthroplasty (PKA) is performed from medial parapatellar arthrotomy without dislocating the patella. Tourniquet is used for every patient if no contraindications exist. Retropatellar fat pad is partially resected if necessary, to ensure adequate surgical visualization. The anterior cruciate ligament is inspected for possible ligament rupture and fibrillation, and lateral and patellofemoral joint compartments are inspected for possible cartilage damage. Patients with lateral OA grade II or greater are excluded from the study at this point. Grade of medial femoral and tibial cartilage loss is verified. All osteophytes are removed from the intercondylar notch, medial margin of the medial condyle, tibial plateau, and patella. PKA instrumentation and implantation are performed according to the cementless Oxford® PKA Microplasty® (Zimmer Biomet, Warsaw, IN, USA) instrumentation system technique. Local infiltration analgesia is used for all patients. The wound is closed in a standard manner. An elastic bandage is applied. Full weight-bearing is allowed immediately after the surgery [21].

### Outcomes

The outcomes of this study consist of objective and patient-reported outcomes. The outcomes will be collected at randomization, preoperative visit, and postoperatively 3, 6, and 12 months. We will follow up the participants thereafter at 2, 5, and 10 years via letter survey.

#### Baseline data

Baseline data is collected at the time of randomization at the outpatient clinic. These include radiographs of the affected knee, weight-bearing mechanical axis, MRI, KOOS_5,_ Lysholm, Oxford knee score, and Osteoarthritis Research Society International (OARSI) performance-based measures. The patients receive an activity watch (Withings Move®, Issy-les-Moulineaux, France), which they are asked to use day and night throughout the trial.

#### Primary outcome measure

The Knee Injury and Osteoarthritis Outcome Score (KOOS) is a validated patient-reported outcome measure (PROM) assessing the outcomes of various knee conditions [22]. The tool consists of five subscales: pain, other symptoms, activities of daily living, function in sports or recreational activities, and quality of life. We will use the KOOS_5_ composite score as the primary outcome measure. Based on clinical experience and literature, there will be only minimal change in pain and PROM scores after 1 year postoperatively [23]. Thus, the primary outcome measure, KOOS_5_ score for HTO vs. UKA is analyzed 12 months postoperatively. A minimal clinically important difference (MCID) of 10 points will be used [24].

#### Secondary outcome measures

Secondary outcome measures are divided into objective (OARSI, length of stay, Withings activity measures, Radiographs [KL and OA progression]), patient-reported (KOOS subscales, pain visual analog scale [VAS], Lysholm, and Oxford knee scores), and adverse events (conversion to TKA, complications [peri- and postoperative], need for revision surgery) outcomes.

In addition to KOOS_5_, additional PROMs are gathered from the Lysholm score (MCID, 4.2 points) [25] and Oxford knee score (MCID, 5 points) [26]. We will also report KOOS subscales individually (ADL, Pain, Symptoms, Quality of life, Sport/Rec) [27].

Average pain for the previous 2 weeks is measured using VAS, which is validated to knee OA pain (MCID, 17 mm) [28, 29].

The OARSI performance-based tests are performed by a physical therapist at baseline and at follow-up visits as shown in the participant timeline in Table 2. This includes 30-s chair stand test (MCID, 2 repetitions), 40-m fast-paced walk test (MCID, 0.2 m/s), stair climb test (MCID, 5.5 s), the timed up and go test (MCID: reduction of 0.8 s), and 6-min walk test (MCID, 20 m increase) [30].

**Table 2:**
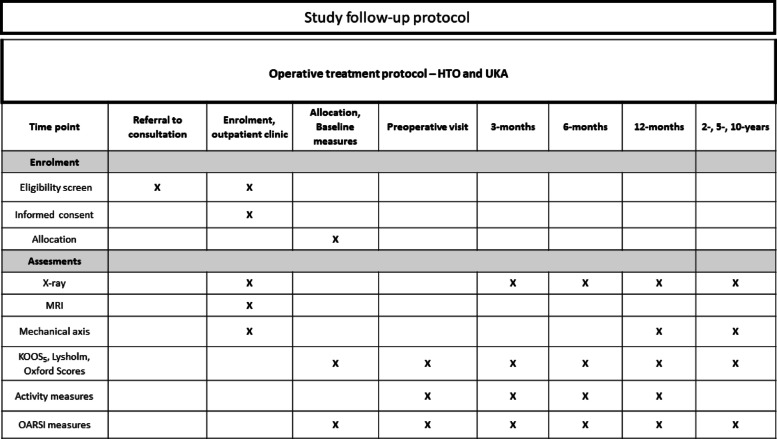
Participant timeline of the trial

After the surgery, the days spent in the hospital will be recorded to follow the early recovery. Early readmissions within 90 days after surgery for any reason are reported for the analysis of possible complications. See safety considerations for the list of complications monitored.

The OA progression is monitored at the follow-up visits using radiographs of the knee at 3, 6, and 12 months after surgery, and the mechanical axis is re-evaluated at 12 months post-randomization. Patients not satisfied with the results of operative treatment (HTO or UKA) can be converted to TKA at any time during the follow-up.

For later determining the MCID of KOOS_5_ in this study, we will include an anchor question to the trial: “If you think about your pain level and daily activities this week, would it be acceptable that your knee would be like this for the rest of your life?”. The answer options are “Yes” or “No.” The proportion of patients reaching the Patient Acceptable Symptom State (PASS) be analyzed from the answers to this question.

We will use the Withings Move electronic watch as an activity tracker. The watches are given to patients right after randomization and the patients are asked to wear them for 24 h per day until the primary time-point of 12 months. The following data are recorded: ID number, date, time, number of steps per day, and sleeping hours. The watch is connected to a secured database and the data is wirelessly transferred and stored.

#### Cost-effectiveness

The consumption of healthcare and social services, used implants, pain medication, and alternative medical services and all related costs will be included in the cost-effectiveness analysis. The costs and benefits will be evaluated against the difference in our primary outcome and in the results of the KOOS quality of life subscale. Used implants, pain medication, and consumption of alternative therapies (i.e., osteopath, chiropractor, naprapath, healer) will be recorded.

The outcome measures are summarized in Table 3.

**Table 3:** Outcome measures

**Primary outcome measure**
1. KOOS_5_ at 12 months postoperatively
**Secondary outcome measures**
1. KOOS subscales (pain, symptoms, activities of daily living, function in sports or recreational activities, and quality of life)
2. Average pain for the last 2 weeks (VAS 0–100 mm)
3. Lysholm and Oxford knee score
4. The 2013 OARSI recommended set of performance-based tests of physical function (i.e., 30-s chair stand test, 40-m fast-paced walk test, stair climb test, the timed up and go test, and 6-min walk test)
5. Days spent in hospital, readmission 90 days
6. Withings Move activity results
7. Radiographic progression of the arthrosis (KL grade mean) at 12 months
8. Changes in mechanical axis at 12 months (degrees from neutral axis)
9. Complications
10. Cost-effectiveness
11. Re-operation
12. Conversion to TKA

### Safety considerations

Adverse surgical complications (deep wound infection, deep venous thrombosis, mechanical failure, post-operative fracture, neurologic complication) and minor complications (superficial wound infection, pain) are collected and reported in the results.

### Sample size

The sample size calculation was performed using G*Power 3.1 and was based on KOOS_5_ as the primary outcome measure in this trial. For the sample size calculation, we used a two-sided α level of 0.05. We assumed the MCID of the KOOS_5_ to be 10 points, [27, 31] with an SD of 15 points. Using these assumptions, the required sample size is 36 per group with 80% power to show a clinically important difference between the treatment methods with a two-sided type I error rate of 5%. With the assumption of 36% lost to follow-up, we decided to include 50 participants per group.

### Allocation

Block randomization will be used in this study. A person not involved in the execution of the trial generates the randomization list using block randomization. The same person prepares sealed envelopes containing the treatment allocation information (HTO/UKA). The block size is not revealed to the study group before analyses. The envelopes are stored in a secure place at the study center. After receiving the informed consent, a surgeon member of the study gives a sealed envelope to the patient containing the treatment allocation information, and the surgery is arranged accordingly.

### Blinding

The physical therapist will be blinded from the treatment allocation when collecting the objective measurements. The patient will be wearing long trousers during the follow-up visits and asked not to reveal the given treatment. The blinding of operative treatment is not possible for the personnel executing the operative treatment nor the patients.

### Statistical analyses

We will conduct all primary and secondary analyses according to the intention-to-treat principle. The Consolidated Standards of Reporting Trials statement will be used in the reporting of the trial results [32].

We will conduct the primary comparison (KOOS_5_ composite score) between the study groups using a mixed-model repeated-measures analysis of variance (MMRM ANOVA) allowing possible data missingness. We will assume data missing at random. Study group and time of assessment will be used as fixed factors and patients will be used as random factors. We are using the model to quantify the treatment effect as the absolute difference between the groups in KOOS_5_ (mean and 95% confidence interval [CI]) and *p*-value at 12 months postoperatively. A two-sided *p*-value of 0.05 will be used to indicate statistical significance.

We will compare secondary outcomes using a similar model where applicable (e.g., KOOS subscales, pain-VAS, Lysholm, Oxford knee score, OARSI). Radiological classification (KL- classification) is used for covariate in post hoc analysis. For categorical response variables, we will analyze the effects using the generalized estimating equations model with the unstructured correlation structure. Secondary outcomes will be considered explanatory and/or exploratory. Thus, multiplicity is not considered a problem. We will report adverse events descriptively.

We plan to perform a sensitivity analysis according to as-treated principle where the patients are analyzed according to their definitive treatment method irrespective of the randomization.

### Data monitoring

Data monitoring committee, interim analyses, or stopping guidelines are not included in this study because both operative treatment options are already in daily practice and the results have been acceptable.

### Harms

All complications and harms are reported in the results section of this study. Major and minor complications are listed in the safety consideration section.

### Protocol amendments

In the case of modification of the study protocol, all changes will be updated to ClinicalTrials.gov.

### Confidentiality

Trial data will be stored in a secure storage at the study center for 10 years after the completion of the study. All data will be handled according to the principles of the GDPR.

### Participant timeline

**Fig. 1:**
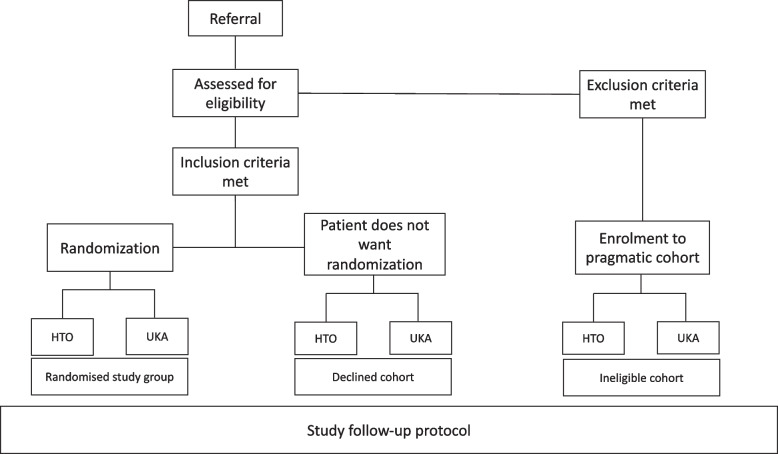
Study flow chart

The participant timeline is represented in Table 2 and the flow chart is in Fig. 1.

### Implementation

The recruitment is done by the surgeon member of the study. After receiving the written consent, the surgeon member opens the envelope, and the patient is then randomized to one of the study groups. A physical therapist does the baseline measures, and the patient receives a written guide for exercise therapy.

### Secondary cohort study

Eligible patients declining randomization are offered to participate in a concurrent observational cohort (“declined cohort”). In addition, patients who are ineligible for the RCT but are treated with either UKA or HTO are asked to take part in a second cohort study (“ineligible cohort”). Those consenting to these cohorts can choose their preferred treatment method after information on both treatment methods is given. Both cohorts are followed up according to the same principles as the participants of the RCT. The results of the declined and ineligible cohorts will be analyzed separately from the RCT.

### Data collection and management

The data is collected by the physical therapist and research nurse using paper forms. The original paper forms are evaluated visually, and missing data is acquired if possible. From the paper forms the data is secured in an electronic archive. Only the research nurse is allowed to access the data during data gathering. In case of missing items in the master data, the original paper forms are reassessed and if needed the patient is contacted.

### Blinded data interpretation

All data collected are interpreted by a blinded scheme. A statistician provides results from each arm of the study labeled A and B. The writing committee then analyzes the results and a consensus on all alternative interpretations is agreed. After a common agreement has been reached, the statistician reveals the randomization code, the correct interpretation is chosen, and the manuscript is finalized [33].

### Ancillary and post-trial care

Patients will be treated during and after the trial with best intention. If malpractice has taken place, patients will not receive any compensation beyond those from the Finnish Patient Insurance Centre.

## Discussion

Both UKA and HTO have been used in clinical practice for treating late-stage unicompartmental knee OA but to our knowledge, there are no previous RCTs comparing UKA with HTO in patients with this condition. In general, HTO has been recommended for younger more active adults with a low-grade unicompartmental OA [9, 15]. However, HTO has been used also in OA patients with higher grade OA [34, 35] and is considered to share indications with UKA in some cases [36]. Thus, we chose to study patients with KL grade III–IV OA.

We will use KOOS_5_ as the primary outcome as we feel that the outcome should be primarily analyzed by the subjective feeling of the patient. A Finnish version of the KOOS score has been appropriately translated and culturally adapted and it has demonstrated good validity and reliability [22]. Additionally, most of our secondary outcomes will be patient-reported as we feel that the most important result of the intervention is the patient’s subjective feeling rather than any objective measure.

As a novel objective measure, a Withings Move activity watch is used for counting the number of steps and hours of sleep per day. This watch has been validated for counting steps and also for analyzing the hours of sleep [37]. We will use the same device in every group for all patients. These objective measures will add to the OARSI performance-based tests used in various OA studies.

### Generalizability

Although OA of the knee is very common, not every OA patient meets the inclusion criteria. The surgical options in this study are very different from the ideological point of view and participants might have a strong preference for one of the treatment modalities, causing participation bias. Therefore, we decided to have a secondary cohort for the declined patients.

### Expectations

We expect that KOOS scores improve significantly after operative treatment during the follow-up. We expect that there is no clinically relevant difference between HTO and UKA treatment in late-stage medial knee OA. Our results will provide high-quality evidence on the surgical treatment options for patients suffering from late-stage medial knee OA.

### Trial status

The present protocol is version 1.2 (2022–12-27). The trial will start in the first half of 2023. We assume to complete the recruitment by the end of 2025.

## Supplementary Information

**Additional file 1.**

## Data Availability

The datasets generated during and/or analyzed during the current study are not publicly available but are available from the corresponding author on reasonable request.
