# Supplementary material for: Unicompartmental knee arthroplasty vs. high tibial osteotomy for medial knee osteoarthritis (UNIKORN): a study protocol of a randomized controlled trial
Source: Trials. 2023 Apr 5;24:256. doi: 10.1186/s13063-023-07263-7 (PMC10074655; doi:10.1186/s13063-023-07263-7)
Supplement: Supplementary file 1 — Additional file 1. [file 13063_2023_7263_MOESM1_ESM.pdf]

Funding disclosure

This statement discloses authors to research funding, including those of persons related to the authors such as spouses, dependent children, or adult children employed by a sponsor of the research, or any other relationship involving the actual or potential sharing of income or assets.

The authors did not have any financial disclosures including the following:

- Leadership Position: Employment or service as an officer or board member for an entity having an investment, licensing, or other commercial interest in the subject matter under consideration
- Consultant/Advisory Role: Arrangements with an entity having an investment, licensing, or other commercial interest in the subject matter under consideration if performed or payments made within two years or anticipated to be paid in the future
- Stock Ownership: Any ownership interest including stock, options, and warrants in any publicly traded or privately held company if the company is an entity having an investment, licensing, or other commercial interest in the subject matter under consideration, except an interest of less than 5% in a publicly traded fund not controlled by the covered individual
- Honoraria: Honoraria for specific speeches, seminar presentations, or appearances when paid to the covered individual by an entity (or agent of the entity) having an investment, licensing, or other commercial interest in the subject matter under consideration and when provided within two years or anticipated to be paid in the future
- Research Funding: Payments associated with the conduct of the clinical research project in question if provided by the trial sponsor or agent of the entity
- Expert Testimony: Provision of expert testimony related to the subject matter under question
- Other Compensation: Trips, travel, gifts, or other in-kind payments not directly related to specific research activities (if not disclosed as related to research related funding) if received from an entity having an investment, licensing, or other commercial interest in the subject matter under consideration and when received within two years of the activity or subject matter in question or anticipated to be received within one year after submission. Aggregate amounts of less than $500 per payor (including its agents) are excluded from disclosure requirements.

Attestation of Investigator Independence/Accountability:
The authors have full access to all study data, take full responsibility for the accuracy of the data analysis, and have authority over manuscript preparation and decisions to submit the manuscript for publication.

Institutional Conflicts of Interest: The authors' academic institutions or employers do not have any financial interest in or a financial conflict with the subject matter or materials discussed in this manuscript.

As corresponding author, I attest that the information on this form is true:

Name (please type or print) Juuso Siren

Signature ______________________ Date: 7.16.2022
